# The Birthday Honours

**Published:** 1910-07-02

**Authors:** 


### The Birthday Honours.

An unusually long list of Birthday Honours this year contains many announcements that are of interest to the medical profession. First and foremost the congratulations of the medical world, and of his many friends outside it, are due to Sir Walter B. Foster on his elevation to the Peerage. The new peer is a very strenuous party politician, but he commands both respect and affection from opponents as well as from those of his own side. No doubt the many honours he has received have been mainly owing to his association with the advanced wing of his party, and his membership of the House of Commons from 1885 to the present year; but it was as Professor of Medicine for twenty-four years that Sir Walter laid the foundations of his subsequent success as Parliamentary Secretary to the Local Government Board in the Parliament of 1892-95. A baronetcy is conferred on Dr. F. H. Champneys, a distinction which crowns a long and honourable career of eminence as an obstetrician. Dr. Champneys is Chairman of the Central Midwives Board, Physician Accoucheur to St. Bartholomew's Hospital, and has been connected with the staff of St. George's and the General Lying-in Hospitals. Seven knighthoods are conferred on members of the profession, though some of these are evidently of political rather than professional intention. Dr. A. H. Downes is senior medical inspector for Poor-law purposes to the Local Government Board. Mr. John Fagan is consulting surgeon to the Belfast Royal Hospital and the Belfast Hospital for Sick Children; he has been for many years inspector of reformatory and industrial schools in Ireland. Mr. George Hastings has been a Lieut.-Colonel of Volunteers. Mr. John Lentaigne is surgeon to the household of the Lord Lieutenant of Ireland and is a well-known hospital surgeon in Dublin; he is also President of the Irish Royal College of Surgeons. Dr. Lunn is the head and founder of the well-known tourist agency, and was for a short time a medical missionary in India before he turned his attention to commerce and politics. Dr. D. C. McVail is a well-known Glasgow physician who has held three professorships in that city, has been physician to the Glasgow Infirmary, and is now Crown member for Scotland of the General Medical Council. Dr. R. M. Simon is also an ex-professor and is physician to the Birmingham General Hospital.

Among the various orders of knighthood which have been bestowed, the most important is the Knight Commandership of the Bath which Inspector-General James Porter, of the Navy Medical Service, receives. The principal medical officers in India and South Africa, respectively Surgeon-General Sloggett, C.M.G., and Surgeon-General O. E. P. Lloyd, V.C., receive the distinction of the C.B. Surgeon-General C. P. Lukis becomes C.S.I.; he is Director-General of the Indian Medical Service and Honorary Surgeon to the Viceroy. Colonel R. Macrae, lately Inspector-General of Civil Hospitals in Bengal, becomes C.I.E. The Viceroy's surgeon, Lieut.-Colonel W. R. Crooke Lawless, is made a Knight. The Kaisar-I-Hind gold medal is awarded to Captain R. McCarrison and Dr. T. L. Pennell; the former is Agency-Surgeon at Gilgit and is best known by his able researches into the causation of the goitres which are common in his district; the latter is a medical missionary whose reputation has spread far beyond the immediate scenes of his labours. The only medical member of the Colonial Services to receive recognition is Dr. A. D. Hodges, the principal medical officer of Uganda, who becomes C.M.G. Lastly, two officers who have worked very hard for the Territorial Medical Service are rewarded: Colonel Andrew Clark by appointment to be Honorary Surgeon to the King, and Colonel J. W. Blandford by a similar position as Physician.

